# DNA hypermethylation of *zygote arrest 1 (ZAR1)* in hepatitis C virus positive related hepatocellular carcinoma

**DOI:** 10.1186/2193-1801-2-150

**Published:** 2013-04-10

**Authors:** Keiko Takagi, Kyoko Fujiwara, Tadatoshi Takayama, Takao Mamiya, Masayoshi Soma, Hiroki Nagase

**Affiliations:** Department of Digestive Surgery, Nihon University School of Medicine, 30-1 Oyaguchi-Kamicho, Itabashi-ku, Tokyo, 173-8610 Japan; Innovative Therapy Research Group, Nihon University Research Institute of Medical Science, Nihon University School of Medicine, 30-1 Oyaguchi-Kamicho, Itabashi-ku, Tokyo, 173-8610 Japan; Division of General Medicine, Department of Medicine, Nihon University School of Medicine, 30-1 Oyaguchi-Kamicho, Itabashi-ku, Tokyo, 173-8610 Japan; Chiba Cancer Center Research Institute, 666-2 Nitona-cho, Chuo-ku, Chiba-shi, Chiba, 260-8717 Japan

**Keywords:** Hepatocellular carcinoma, Hepatitis C virus, Methylation

## Abstract

**Background:**

Hepatocellular carcinoma (HCC) is one of the most common human malignancies in the world, and its prognosis is generally poor. Epigenetic alteration such as DNA methylation has been shown to be important in the development of human cancers including HCC. Here, we analyzed the methylation status of *ZAR1*, which has been reported to be aberrantly methylated in a few human cancers.

**Methods:**

We investigated the methylation status of *ZAR1* in 88 HCV-positive HCC and matched nontumorous liver tissue samples and 4 normal liver tissue samples used as a control using MassARRAY EpiTYPER. Further statistical analysis was performed to determine the relationship between methylation level and patient clinicopathological features and prognosis.

**Results:**

CpG islands in *ZAR1* exon 1 showed a higher methylation level in all 88 HCC than in nontumorous tissues. The hypermethylation group, whose cancer tissues showed a twofold or higher methylation level compared with nontumorous tissues, showed a significantly higher serum AFP (*p* = 0.018) and lower serum albumin (*p* = 0.001) and single rather than multiple tumors (*p* = 0.031) compared with the hypomethylation group. Multivariate regression analyses were performed to identify which of the following factors were the predictors of the hypermethylation group: serum albumin, AFP, and tumor multiplicity. This study showed that patients who had *Zar1* hypermethylation in the HCC tissues had a significantly lower serum albumin level than those in the hypomethylation group (*p* = 0.007).

**Conclusion:**

Although it is still unknown how *ZAR1* hypermethylation affects HCC development, it could be a potential marker to detect HCV-related HCC.

## Background

Hepatocellular carcinoma (HCC) is one of the most common human malignancies in the world. Despite the fact that diagnosis and treatment have been established for HCC, the prognosis is generally poor. Even after liver resection, 25%, 50%, and 80% of HCC patients suffer relapses within 1, 2, and 5 years, respectively. Although the incidence of nonBnonC-HCC has recently shown an increasing trend, there still are many HCV-positive HCC patients in Japan (Ikai et al. 2007; Makuuchi et al. 1998). Although the molecular mechanisms of its pathogenesis remain unclear, epigenetic alteration such as DNA methylation has been shown to play an important role in the development of human cancers (Shinojima et al. 2010; Kawashima et al. 2012; Watanabe et al. 2011). In addition, aberrant DNA methylation of promoter CpG islands of many genes has been reported for HCC. Methylation of promoter CpG islands of the tumor suppressor genes has been reported. Genes such as *p16*, *p15*, *GSTP1*, *SOCS-1*, *RASSF1A*, and *APC* play an important role in HCC development (Narimatsu et al. 2004; Yang et al. 2003; Tischoff & Tannapfel 2008). These genes are involved in the regulation of the cell cycle, xenobiotic metabolism, suppression of cytokine signaling, apoptosis, and cell migration. Most DNA methylation studies have concentrated on genes mainly inactivated by hypermethylation of normally unmethylated CpG islands containing promoter regions. However, some researchers have shown that CpG hypermethylation in nonpromoter regions is sometimes associated with the upregulation of gene expression (Smith et al. 2007). Shinojima et al. and Watanabe et al. recently reported aberrant methylation in candidate human genomic regions that was identified through the analysis of skin tumor-specific differentially methylated regions in mouse models using the restriction landmark genomic scanning (RLGS) method. They examined differentially methylated regions in the exon of *zygote arrest 1* (*ZAR1*) that had never been linked to aberrant methylation in melanomas and brain tumors (Shinojima et al. 2010; Watanabe et al. 2011). Here, we detected aberrant methylation of the nonpromoter region of *ZAR1* in hepatitis C virus-positive HCC tissues compared with nontumorous liver and metastatic liver cancer tissues.

## Results and discussion

**Figure 1:**
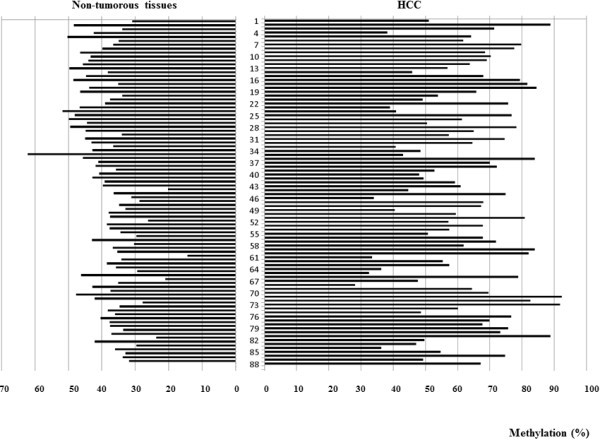
**Average methylation level of all CpG sites in*Zar1*in each individual.** A total of 67 of 88 cases showed a significantly higher methylation level in HCC than in matched nontumorous tissues (*p* < 0.05). Data are shown as mean ± SD.

**Figure 2:**
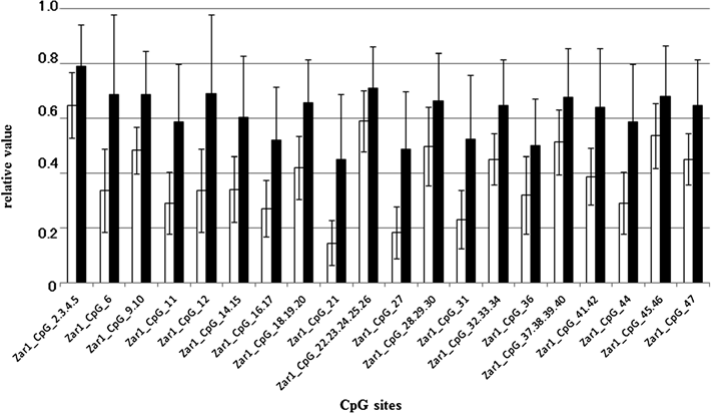
**Average methylation level of each CpG site in*Zar1*in HCC and nontumorous liver tissues.** Open bars indicate nontumorous tissues, and filled bars indicate HCC tissues. All CpG sites showed a significantly higher methylation level in HCC than in nontumorous tissues (*p* < 0.05). Data are shown as mean ± SD.

The methylation level of CpG islands in *ZAR1* exon 1 (RLGS spot 5D52) was analyzed in 88 HCV-positive HCC and 4 normal liver tissues, which were circumjacent to colon cancer liver metastasis. The average methylation level of all CpG sites in *Zar1* in each individual was calculated, and 67 of 88 cases showed a significantly higher methylation level in HCC than in nontumorous tissue (*p* < 0.05) (Figure 1). The average methylation level of each CpG site in *Zar1* of all 88 cases are shown in Figure 2. All CpG sites showed a significantly higher methylation level in HCC than in the matched nontumorous tissues (*p* < 0.05). Because it is already known that HCV-positive nontumorous tissues undergo some epigenetic changes, we also compared the methylation level of *Zar1* in normal liver tissues with that in HCV-positive nontumorous tissues; however, no difference was found between the tissues (data not shown).

The hypermethylation group was defined as the sample set in which patients’ cancer tissues showed a twofold or higher methylation level of *ZAR1* compared with nontumorous liver tissues. A total of 22 and 66 patients were enrolled in hypermethylation hypomethylation groups, respectively. When the background clinicopathological features of the hypermethylation and hypomethylation groups were compared using univariate analysis, there were significant differences in levels of serum AFP (*p =* 0.018) and albumin (*p =* 0.001) and tumor multiplicity (*p =* 0.031) (Table 1). Multivariate regression analyses were performed to identify which of the following factors were the predictors of hypermethylation: serum albumin, AFP, and tumor multiplicity. The presence of low serum albumin level increased the risk for hypermethylation (*p* = 0.007) (Table 2).

**Table 1:** **Comparison of clinicopathological features between the 2 groups**

Variable		Hypermethylation group n = 22 (%)	Hypomethylation group n = 66 (%)	***P***
Gender	M	17 (77.3)	58 (87.9)	0.225
	F	5 (22.7)	8 (12.1)	
Age (y)^a^		65.8 (50.0-78.0)	68.5 (50.0-78.0)	0.07
ICG15R (%)	< 10	6 (27.3)	26 (39.4)	0.306
	≧10	16 (72.7)	40 (60.6)	
Alb (g/dl)	< 3.5	9 (40.9)	6 (9.1)	0.001
	≧3.5	13 (59.1)	60 (90.9)	
AST (U/l)	< 40	7 (31.8)	21 (31.8)	1.000
	≧40	15 (68.2)	45 (68.2)	
ALT (U/l)	< 40	9 (40.9)	27 (40.9)	1.000
	≧40	13 (59.1)	39 (59.1)	
T.Bil (mg/dl)	< 1.2	20 (90.9)	59 (89.4)	0.839
	≧1.2	2 (9.1)	7 (10.6)	
AFP (ng/ml)	< 20	8 (36.4)	42 (63.6)	0.018
	≧20	14 (63.6)	24 (36.4)	
non-LC		10 (45.5)	41 (62.1)	0.170
LC		12 (54.5)	25 (37.9)	
Vascular invasion	(+)	10 (45.5)	43 (65.2)	0.102
	(−)	12 (54.5)	23 (34.8)	
Tumor multiplicity	single	22 (100)	54 (81.8)	0.031
	multiple	0 (0)	12 (18.2)	
Liver damage	A	15 (68.2)	38 (57.6)	0.379
	B	7 (31.8)	28 (42.4)	
Recurrence	(+)	19 (86.4)	52 (78.8)	0.436
	(−)	3 (13.6)	14 (21.2)	

^a^Data are given as median (range).

ICG15R, indocyanin green retention rate at 15 min; Alb, albumin; AST, aspartate aminotransferase; ALT, alanine aminotransferase; T.Bil, total bilirubin; AFP, alpha fetoprotein; LC, liver cirrhosis.

**Table 2:** **Independent risk factors predicting hypermethylation in multivariate logistic regression analysis**

	Odds ratio	95% CI	***p***
Alb (g/dl) < 3.5	5.921	1.643-21.335	0.007

These results demonstrated that DNA methylation levels of *ZAR1* were significantly different between HCV-related HCC and nontumorous tissues. Most studies on the analysis of DNA methylation in cancer genomes have focused on promoter CpG island methylation and examined only restricted sets of CpG sites in their target regions. To identify genome-wide aberrant methylation in mouse skin cancer tissues, we previously performed RLGS analysis and then confirmed the results using MassARRAY EpiTYPER. The RLGS method can semiquantitatively detect the change in methylation level of NotI in global genomic regions, including the regions outside the promoter. MassARRAY EpiTYPER can quantitatively analyze the methylation level of CpG sites by base-specific cleavage in combination with MALDI-TOF-MS detection. Using these methods, we were able to detect a differentially methylated region in the exon of *ZAR1* that had never been identified as an aberrantly methylated region in clinical HCC tissue samples (Shinojima et al. 2010; Smiraglia et al. 2007). Our findings clearly showed that aberrant hypermethylation of the *ZAR1* nonpromoter region was common in many HCV-related HCC tissues.

*ZAR1* is an ovary-specific maternal factor that has critical importance in the initiation of embryogenesis (Wu et al. 2003). Mouse and human *ZAR1* protein contain 361 and 424 amino acids, respectively, and have 59% similarity in amino acid sequence. Although the *ZAR1* protein is not a member of any characterized protein family, it contains an atypical plant homeodomain (PHD) finger in its C-terminus. PHDs are found in two major classes of proteins, such as transcriptional activators, repressors, or cofactors and subunits of complexes that modulate chromatin (Wu et al. 2003). *ZAR1* may be a transcriptional regulator, and the conserved *ZAR1* C-terminus is probably important for its function. Shinojima et al. analyzed malignant melanomas and reported that tumor-specific methylation of CpG islands in *ZAR1* that were located downstream from the promoter region was associated with an increased expression level of the *ZAR1* transcript. Although they transfected the full-length *ZAR1* cDNA plasmid into normal melanocytes and also performed knock down of *ZAR1* expression in melanoma cells using siRNA, no differences were observed in either the cell growth rate or the morphology of the transfected cells (Shinojima et al. 2010). In the present study, the hypermethylation group, whose cancer tissues showed a twofold or higher methylation level compared with nontumorous liver tissues, showed a significantly higher level of serum AFP (*p* = 0.018) and lower level of serum albumin (*p* = 0.001) compared with the hypomethylation group, using univariate analysis. In addition, all the patients in the hypermethylation group developed only single, but not multiple, tumors (*p* = 0.031). Multivariate regression analyses were performed to identify the predictors of the hypermethylation group. The patient with low serum albumin level had an increased risk for hypermethylation (*p* = 0.007). In general, it was already known that a high serum AFP level and low serum albumin level are associated with development and poor prognosis of HCC (Tateyama et al. 2011; Ueno et al. 2002). Albumin is a group of proteins produced mainly in the liver. Because the method for measurement of albumin concentration is simple and easy, the serum albumin level has been used as a prognostic factor following liver resection. However, owing to the long half-life of albumin, it is not a sensitive marker to detect events changing over a short period (Shirabe et al. 2011; Horino et al. 2012). In this study, the mean values of methylation level of all CpG sites in HCC tissues were higher, and the presence of low serum albumin level increased the risk for hypermethylation (*p* = 0.007). For these reasons, it is suggested that *ZAR1* hypermethylation could be a novel and good marker to detect HCC and also could be a potential marker for HCC prognosis.

*ZAR1* mRNA expression was not detected in any of the tissue specimens in this study. There is a possible explanation for this result. The aberrant hypermethylation of *ZAR1* may be the result of a secondary effect of the tumorigenesis process, or the methylation level of *Zar1* exon 1 may be involved in the regulation of other genes distant from *Zar1*. Further studies are needed to understand the role of hypermethylation of the *Zar1* genomic region in HCC development.

## Conclusions

This study showed that the methylation level of the *Zar1* genomic region is higher in HCV-related HCC than in nontumorous tissues and that patients with *Zar1* hypermethylation had a significantly lower serum albumin level than those from the hypomethylation group. Although it is still unknown how *ZAR1* hypermethylation affects HCC development, it could be a marker for HCV-related HCC and also could be a potential marker for predicting HCC prognosis.

## Methods

### Patients and tissue samples

Eighty-eight hepatitis C virus-positive HCC and matched nontumorous tissue and four normal liver tissues, which were circumjacent to tissues of metastatic liver cancer, were obtained from the Nihon University Hospital, Japan, from 1995 to 2010. Informed consent was obtained from all patients. All liver tissue specimens were immediately frozen in liquid nitrogen and stored at −80°C until use. Nontumorous tissues were excised with sufficient margins from the cancer. This study was approved by the Institutional Review Boards of Nihon University School of Medicine.

### DNA extraction and bisulfite treatment

DNA was extracted using the standard phenol–chloroform purification method or a QIAamp DNA Mini Kit (Qiagen, Valencia, CA, USA). Bisulfite modification of DNA was performed using an EZ DNA Methylation Gold Kit (Zymo Reseach, Orange, CA, USA). Bisulfite-treated genomic DNA was amplified using HotStar Taq Polymerase (Qiagen) for 15 min at 94°C followed by 45 cycles of 20 s at 94°C, 30 s at 56°C, and 1 min at 72°C, with a 3-min final extraction at 72°C. The polymerase chain reaction (PCR) products obtained were subjected to gel electrophoresis and then measured using Sequenom MassARRAY.

### Quantitative methylation analysis using base-specific cleavage and matrix-assisted laser desorption/ionization time-of-flight mass spectrometry (MALDI-TOF-MS)

A Sequenom MassARRAY quantitative DNA methylation analysis was performed (Ehrich et al. 2005) using the MassARRAY Compact System. This system is based on mass spectrometry (MS) for the detection and quantitative analysis of DNA methylation using homogeneous MassCLEAVE (hMC), base-specific cleavage, and matrix-assisted laser desorption/ionization time-of-flight MS (MALDI-TOF MS) (Ehrich et al. 2005). The *ZAR1* primer was designed using Methyl Primer Express® Software to span the closely adjacent DMRs or the GC rich region, as indicated. The primers used for the quantitative *ZAR1* methylation analysis were designed to anneal to the 374-bp bisulfite-treated fragment for the MassARRAY EpiTYPER (Sequenom, San Diego, CA, USA). The reverse primer has a T7-promoter tag for *in vitro* transcription (5^′^-cagtaatacgactcactatagggagaaggct-3^′^), and the forward primer is tagged with a decamer to balance the temperature (Tm) (5'-aggaagagag-3^′^). These primers were purchased from Operon (Tokyo, Japan). PCR amplification was performed using HotStar Taq Polymerase (Qiagen) in a 5-μl reaction volume using PCR primer at a final concentration of 200 nM and 1 μl of bisulfite-treated DNA (~20 ng/ml). After treatment with shrimp alkaline phosphatase, 2-μl aliquots of the PCR products were used as a template for *in vitro* transcription and RNase A cleavage for the T-reverse reaction, as described in the manufacturer’s instructions (Sequenom hMC). The samples were desalted and spotted on a 384-pad SpectroCHIP (Sequenom) using a MassARRAY nanodispenser (Samsung, Seoul, Korea) followed by spectral acquisition on a MassARRAY Analyzer Compact MALDI-TOF MS (Sequenom). The resultant methylation calls were analyzed using EpiTYPER software v1.0 (Sequenom) to generate quantitative measurements for each CpG site or an aggregate of multiple CpG sites. A standard curve of quantitative DNA methylation analysis was constructed using 0%, 50%, and 100% methylated samples. The amplified DNA was not methylated at all in any of the CpG sites and was used as unmethylated (0%) control. M.SssI double-treated DNA was prepared according to the manufacture’s protocols (New England Biolabs, Ipswich, MA, USA) at 37°C for 1 h followed by inactivation at 65°C for 20 min for 100% methylation samples. By mixing equal amounts of 0% and 100% control samples, the 50% methylation samples were prepared. The standard curve was fitted, and methylation levels were adjusted and quantified.

### Statistical analysis

Comparison of clinicopathological features between the hypermethylation and hypomethylation groups was analyzed by Fisher’s exact test or Mann–Whitney *U* test. Methylation levels of HCV-related HCC and metastatic liver cancer tissues were compared using the *t*-test. Statistical analysis was performed using SPSS version 15.0 (SPSS Inc., Chicago, IL, USA). Differences were considered significant at *p* < 0.05.

## Abbreviations

HCC: Hepatocellular carcinoma
RLGS: Restriction landmark genomic scanning
ZAR1: Zygote arrest 1.
